# High expression of CCNB1 driven by ncRNAs is associated with a poor prognosis and tumor immune infiltration in breast cancer

**DOI:** 10.18632/aging.204253

**Published:** 2022-08-29

**Authors:** Hongtao Fu, Kun Li, Shui Wang, Yuming Li

**Affiliations:** 1 Department of Breast Surgery, Jiangsu Province Hospital, The First Hospital Affiliated Hospital with Nanjing Medical University, Nanjing 210029, China; 2 Department of emergency, Changsha Central Hospital, The Affiliated Changsha Central Hospital of Hengyang Medical School, University of South China, Changsha 410004, China; 3 Department of Traditional Chinese Medicine and Western Medicine, Hunan Cancer Hospital, The Affiliated Cancer Hospital of Xiangya School of Medicine, Central South University, Changsha 410006, China

**Keywords:** biomarker, bioinformatics analysis

## Abstract

Backgrounds: Breast cancer (BC) is the most frequent cancer diagnosed in women throughout the world. The purpose of this study was to explore new biomarkers for breast cancer diagnosis. CyclinB1 (CCNB1) is found in abundance in a wide range of human malignancies.

Material and Methods: We evaluated the transcriptional, survival data and expression levels in tissue data of CCNB1 in patients with breast cancer from the Gene Expression Omnibus (GEO), The Cancer Genome Atlas (TCGA), The Human Protein Atlas (THAP) and Genome Tissue Expression (GTEx) database. A series of in silico analyses were used to investigate at noncoding RNAs (ncRNAs), gene ontology (GO) annotation analysis, Kyoto Encyclopedia of Genes and Genomes (KEGG), and the Protein-Protein Interaction (PPI) network. A quantitative real-time polymerase chain reaction (qRT-PCR) was used to validate CCNB1 in BC cell lines.

Results: CCNB1 expression was higher in BC tissues than in normal breast tissues. It was significantly related to survival time, tumor mutation burden (TMB), methylated, immune cell infiltration, and the expressed in estrogen receptor (ER) (−), lymphnode (+), and p53 (+) groups in BC. Moreover, The AC026401.3/CCNB1-miR-139-5p axis was discovered as the most promising upstream ncRNA-related pathway of CCNB1 in BC.

Conclusion: CCNB1 can be used as an independent predictive factor for BC, indicating that this would be a target for highly precise therapy and a biomarker for the disease.

## INTRODUCTION

Breast cancer (BC) is one of the leading causes of mortality in women throughout the world [1]. Many academics are working to gain a better knowledge of BC’s carcinogenic pathway and to find novel biomarkers. Finding new biomarkers of disease progress and the signal pathway is very important for finding a more effective diagnosis and treatment strategies [2, 3]. CyclinB1 (CCNB1) is a highly conserved family of cyclins that is detected in nearly all human tissues and many of cancer types [4–6]. CCNB1 forms MPF with p34 and is involved in cell cycle regulation [7], and it has also been suggested that CCNB1 is related in the epithelial-mesenchymal transition (EMT) and metastatic processes [8].

The expression of CCNB1 in 33 different cancer types was extracted from The Cancer Genome Atlas (TCGA) and Genome Tissue Expression (GTEx), together with normal tissue transcriptome data, and the expression of CCNB1 in 33 types of cancer was analyzed. Then, we analyzed the expression of CCNB1 in normal breast tissue and BC tissue, and the correlation of CCNB1 with tumor mutation burden (TMB), methylated, and immune cell infiltration in BC. In addition, 347 BC samples were obtained from the GSE4922 of Gene Expression Omnibus (GEO). After independent prognostic analysis and clinical correlation analyses, CCNB1 was selected for further analysis. A quantitative real-time polymerase chain reaction (qRT-PCR) was used to validate CCNB1 in BC cell lines.

We first investigated the regulation of CCNB1 by noncoding RNAs (ncRNAs) including microRNAs (miRNAs) and long noncoding RNAs (lncRNAs). We applied the limma software package with standard data processing function to analyze differentially expressed genes (DEGs), and made differential gene correlation heat map and gene interaction map. The enrichment analysis of Gene Ontology (GO) terms Kyoto and Encyclopedia of Genes and Genomes (KEGG) was then performed. String was used to build and visualize the protein-protein interaction (PPI) network and target gene subnetwork in the Cytoscape program.

The above analysis of bioinformatics will greatly deepen the understanding of the mechanism of CCNB1 in BC.

## METHODS

### Data collection

GSE4922 was acquired from the GEO database, which includes gene expression and clinical data on 347 BC patients. The DEGs of BC samples are compared to normal samples using R’s limma software package. The following criteria were used to define DEGs: adjusted *P* < 0.05 and | logfc | > 0.1. For Pan-cancer analysis, 33 kinds of tumor and normal samples data from TCGA (https://cancergenome.nih.gov/) and GTEx database (https://commonfund.nih.gov/GTEx) were collected using R's ggpubr package (version 3.6.3). The PAM50 procedure was used to classify BC and normal samples into subtypes HER2, Basal like, luminal A, luminal B, and normal [9, 10].

### Pan-cancer expression of the target gene

The expression of the target gene was extracted from the transcriptome data of 33 distinct types of cancer and normal tissues in TCGA database to find out which cancers and their corresponding normal tissues besides cancer had a significant differential expression of the target gene.

### Analysis of TMB, DNA methylation, immune cell infiltration and immunohistochemistry

CCNB1 was compared with 33 distinct types of cancer data in the downloaded TCGA and GTEx data to explore the relationship between the CCNB1 and TMB, and the connection between the CCNB1 and immune cells in BC tissue. The RNAseq data in the medium level 3 HTSeq-FPKM format and the illumina human DNA methylation 450 DNA methylation data and BC DNA methylation data were acquired from TCGA (Spearman correlation coefficient r = −0.140, *P* < 0.001 as the cut-off criterion). The expression levels of CCNB1 in normal breast tissues and BC tissues were validated utilizing Human Protein Atlas (THAP) database (https://www.proteinatlas.org/).

### Survival analysis filter, clinical correlation analysis and independent prognosis analysis

The Kaplan-Meier (KM) and Cox regression method were used to filter, brush, and identify genes with significant differences in this survival evaluation. The set filtering criteria must satisfy KM < 0.05 and Cox value < 0.05. HR > 1 suggested that the gene was upregulated in BC, while HR < 1 suggested that the gene was downregulated in BC. After that, the clinical correlations of these genes with ER, lymph node, p53, age, and tumor size were analyzed, and the genes with the highest connection being chosen for further investigation. The survival time and survival state were extracted, and the gene was visualized by independent prognosis analysis survival analysis.

### Survival analysis

Survival analysis and expression validation of CCNB1 using KM analysis combined with Cox regression were carried out by using the survival and survminer package by R (version 3.6.3).

### Identification of DEGs, function and pathway enrichment analysis

Based on CCNB1 expression, BC cases were separated into low and high groups, with the median expression serving as the cut-off value. Specifically, BC cases were placed in the high group if their CCNB1 expression was higher than the median, and in the low group if their CCNB1 expression was lower than the median. The DEGs were identified by using *P* < 0.05 and | logfc | > 0.1 as the cut-off criterion. High expression samples and low expression samples were used to divide the target genes into two categories.

### Cluster profiler

The Gene Ontology (GO) pathway analysis of DEGs were identified using the R’clusterprofile package. The KEGG pathway analysis of DEGs was predicted using the web program Metascape (https://metascape.org/gp/index.html#/main/step1). The KEGG signaling pathway analysis was carried out by using internet program KEGG (https://www.genome.jp/kegg/) [11].

### Gene mapping and PPI network construction, module analysis of target gene

The first 20 most significant genes were analyzed according to their interaction. The PPI information was then evaluated using string (search tool) that was used to retrieve the interaction genes. We constructed a PPI network of DEGs by string and applied a confidence score ≥ 0.4 as the cut-off criteria to examine the relevant link between these DEGs. The PPI subnet centered on CCNB1 is then graphically generated using the Cytoscape program.

### Candidate miRNA and lncRNA prediction

To find CCNB1 upstream binding miRNAs, we applied miRWalk and miRMap. For the following studies, only the forecasted miRNAs that occurred often in the two algorithms indicated above were used. These projected miRNAs were considered CCNB1 candidate miRNAs.

StarBase (http://starbase.sysu.edu.cn/) is a database dedicated to miRNA research [11]. In BC, starBase was set up to do miRNA-CCNB1, lncRNA-miR-139-5p, or lncRNA-CCNB1 expression correlation analysis. StarBase was used to explore at the expression of miR-139-5p in BC and normal controls. In addition, starBase was utilized to identify candidate lncRNAs that could bind to miR-139-5p. The association between lncRNA expression and CCNB1 was assessed by Spearman correlation.

### Cell culture

MCF-7 (Luminal A), BT474 (Luminal B), SKBR3 (Her2+, i.e., Her2 enriched), MDA-MB-231 (Basal like) and MCF 10A cell lines (Normal) [12] were from the Chinese Academy of Science (Shanghai, China). The three cell lines were cultured at 37°C and 5% CO^2^ in a sterile cell culture incubator. The cell lines except MCF-7 were cultured using MEM (Procell, Wuhan, China) containing fetal bovine serum (FBS, Procell, Wuhan, China) and penicillin (100 U/mL) and streptomycin (100 μg/mL) (P/S, Procell, Wuhan, China) to make up a concentration of 10% FBS and 1% P/S. MCF-10A was cultured with the following medium: DMEM+5% HS+20 ng/ml EGF+0.5 μg/ml hydrocortisone+10 μg/ml insulin+1% NEAA+1% P/S.

### qRT-PCR

Total RNA was isolated using RNA-easy Isolation Reagent (Vazyme), and cDNA was generated with HiScript^®^ III SuperMix (Vazyme) according to the kit’s instructions. qRT-PCR was performed in a StepOnePlus (Applied Biosystems) device (Vazyme) using ChamQTM SYBR^®^ qPCR Master Mix. The specific primers used for qRT-PCR are listed in Table 1. Glyceraldehyde-3-phosphate dehydrogenase (GAPDH) was used as an internal reference gene. CCNB1 was compared and analyzed by 2−ΔΔct method.

**Table 1:** Summary of the oligonucleotide primer sequences.

**Gene/LncRNA**	**Forward primer**	**Reverse primer**
GAPDH	GTCTCCTCTGACTTCAACAGCG	ACCACCCTGTTGCTGTAGCCAA
CCNB1	CCGCTCGAGCGGATGGCGCTCAGGGTCACT	CGCGGATCCGCGTGCCTTTGTCACGGCCTT

### Availability of data and materials

All data and materials used in this work were publicly available and also available based on request.

## RESULTS

### CCNB1 expression in pan-cancer

**Figure 1:**
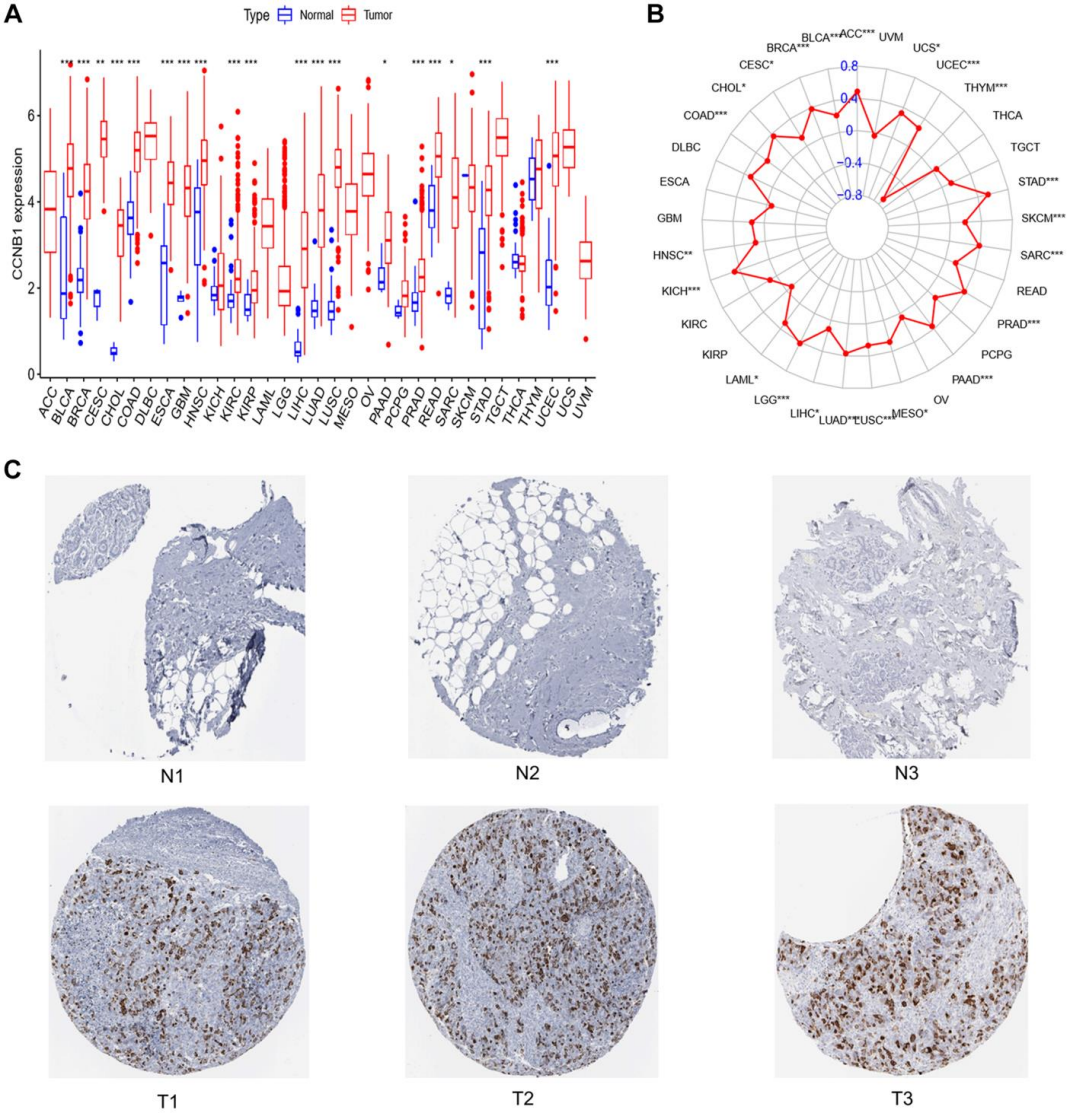
**Expression of CCNB1 in normal breast tissue and tumor tissue.** (**A**) Expression of CCNB1 in pan cancers. CCNB1 expression was shown to be higher in cancer tissues than in normal tissues in the TCGA study. (**B**) TMB of CCNB1 in pan cancer. A positive association with cancer is shown by a red dot bigger than 0; a negative correlation with cancer is indicated by a red dot less than 0. (**C**) The THPA website was used to examine the expression of the CCNB1 protein in BC specimens and noncancerous breast tissue. Three images of malignant and non-cancerous breast tissues were shown. Abbreviations: N: normal; T: tumor; TCGA: The Cancer Genome Atlas; THPA: The Human Protein Atlas; TMB: tumor mutation burden. ^*^P < 0.05, ^**^P < 0.01, ^***^P < 0.001.

The transcriptome data of 33 types of cancer and normal tissues in the TCGA database was used to extract CCNB1 expression. CCNB1 has been discovered to be strongly expressed in 19 cancer types, including BC, while low expressed in normal tissues (Figure 1A).

### Analysis of TMB, immunohistochemistry and immune cell infiltration of CCNB1

**Figure 2:**
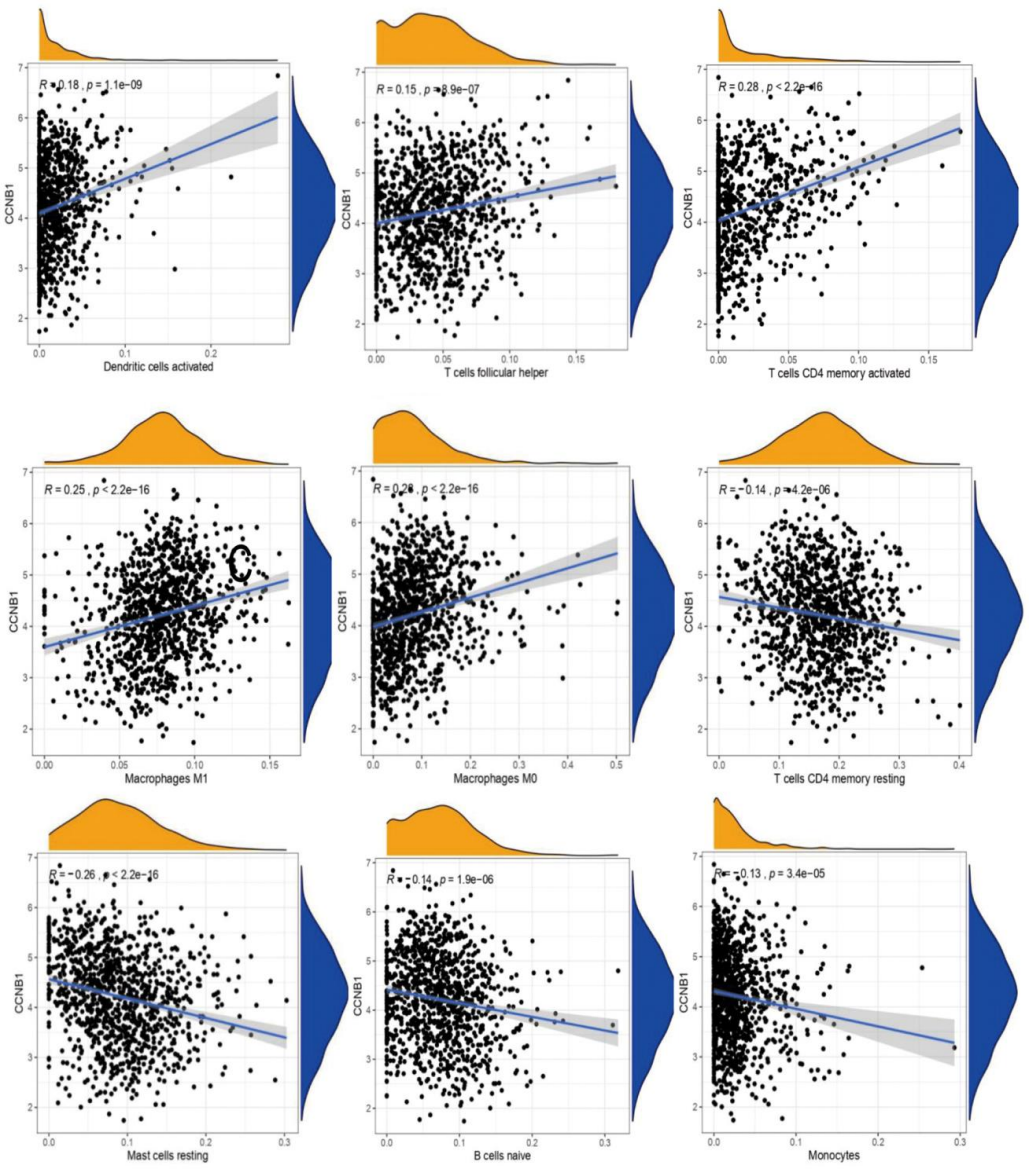
**The correlation between CCNB1 and immune cell infiltration.** Each point (in black) in the figure represents a sample. The abscissa is the content of an immune cell, and the expression of a gene in the ordinate. R > 0 suggests that the gene is positively correlated with the content of an immune cell, R < 0 suggests that the gene is negatively correlated with the content of an immune cell.

In BC, CCNB1 expression was positively correlated with TMB (Figure 1B). In the analysis of immune cell infiltration, the expression of CCNB1 was positively correlated with the expression of Macrophages M0, Macrophages M1, Dendritic cells activated, T cells follicular helper, T cells CD4 memory activated, it was negatively correlated with the expression of B cells naive, Mast cells resting, T cells CD4 memory resting, Monocytes (Figure 2). Eventually, the upregulation of CCNB1 in BC tumor tissues was confirmed by immunohistochemistry in the Human Protein Atlas (THPA) database (Figure 1C).

### Survival analysis

**Figure 3:**
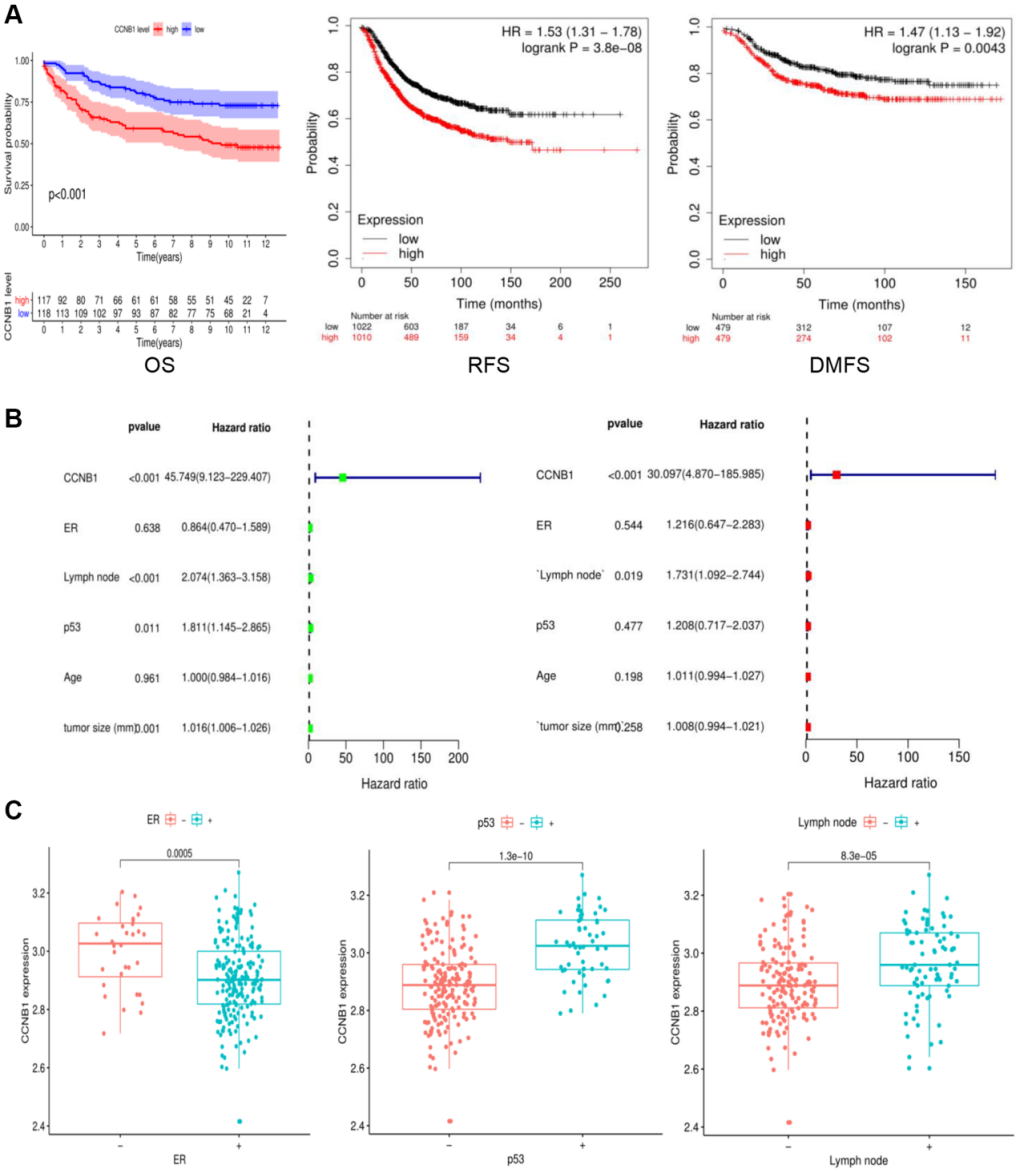
**Survival and clinical association of CCNB1 as an independent prognostic factor in BC.** (**A**) The survival analysis was performed for OS, RFS and DMFS. The CCNB1 high expression group had a considerably shorter survival duration than the CCNB1 low expression group. (**B**) Univariate and multivariate analyses of factors related to survival. (**C**) Clinical characteristics associated with CCNB1. It can be seen that CCNB1 is highly expressed in ER (−), lymphnode (+), and p53 (+) groups. Abbreviations: OS: overall survival; RFS: recurrence free survival; DMFS: distant metastasis free survival.

The CCNB1 high expression group had a substantially lower survival time (Including: OS, overall survival; RFS, recurrence free survival; DMFS, distant metastasis free survival) than the CCNB1 low expression group, demonstrating that CCNB1 expression in BC was adversely linked with survival time (Figure 3A). CCNB1 was statistically significant (*P* < 0.001) in both univariate and multivariate analyses of variables linked to survival, indicating that it may be considered as an independent prognostic factor for BC (Figure 3B).

### Independent prognostic analysis, clinical correlation analysis and single gene selection

BRIP1, CCNB1, CDT1, CENPW, CSRNP3, DIAPH3, ENKD1, LINC00472, LOC101928767, SPC24, SYCP3 are the genes with the significant difference, according to survival analysis filtering (Table 2). Analysis of the clinical associations of these genes with ER, lymph node, p53, age, and tumor size revealed a high correlation between the CCNB1 with ER, lymph node, p53, and tumor size (Table 3). It can be seen that CCNB1 is highly expressed in ER (−), lymphnode (+), and p53 (+) groups (Figure 3C).

**Table 2:** Independent prognostic analysis: Results of KM analysis and Cox regression.

**Gene**	**KM**	**HR**	**HR.95L**	**HR.95H**	**Cox *P* value**
BRIP1	0.000567	15.50514	3.062195	78.5089	0.000925
CCNB1	0.000112	30.93142	6.327355	151.2089	2.25E-05
CDT1	7.79E-05	33.65832	7.249179	156.2774	7.17E-06
CENPW	1.45E-05	17.30945	3.554348	84.29595	0.000415
CSRNP3	0.000105	0.017295	0.002174	0.137595	0.000126
DIAPH3	8.77E-05	31.08617	5.217891	185.1993	0.00016
ENKD1	0.000398	11.88679	2.820575	50.09469	0.000744
LINC00472	0.000572	0.048839	0.00956	0.249506	0.000285
LOC101928767	0.000292	0.161909	0.055285	0.474169	0.000897
SPC24	0.00025	17.2094	4.938437	59.97111	7.92E-06
SYCP3	0.00033	0.262424	0.144904	0.475256	1.01E-05

KM analysis and Cox regression were used in this survival analysis. The difference between KM < 0.05 and Cox value < 0.05 was statistically significant. HR > 1 suggested that the gene was highly expressed in primary invasive breast cancer, while HR < 1 suggested that the gene was low expressed in primary invasive breast cancer. Abbreviation: KM: Kaplan-Meier.

**Table 3:** Clinical correlation analysis.

**ID**	**ER**	**Lymphnode**	**p53**	**Age**	**Tumor size (mm)**	**SigNum**
CCNB1	0.000495	8.25E-05	1.28E-10	0.931491	0.001463	4
CDT1	9.99E-08	0.004014	6.84E-09	0.728761	0.010564	4
SPC24	9.98E-06	0.013015	3.30E-13	0.796478	0.043895	4
BRIP1	7.06E-05	0.001215	3.21E-08	0.90619	0.125911	3
CENPW	6.53E-09	0.000669	3.54E-17	0.794988	0.090089	3
CSRNP3	0.449172	0.000744	0.001321	0.733847	0.043708	3
DIAPH3	0.000349	0.00852	1.09E-06	0.605309	0.194739	3
LINC00472	9.84E-05	0.000575	1.34E-07	0.065608	0.34718	3
SYCP3	0.182944	0.000465	0.011033	0.106715	0.069444	2
ENKD1	0.345043	0.89559	0.995479	0.728761	0.178342	0
LOC101928767	0.065018	0.745138	0.056655	0.130608	0.486453	0

Table 3 gene and ER, lymphnode, p53, age, tumor size four single factor clinical correlation analysis, Signum is the number of genes in Table 3 and the above single factor correlation. There was significant correlation between CCNB1 and ER, lymphnode, p53, age, tumor size. Abbreviations: ER: estrogen receptor; PR: progesterone receptor; HER2: human epidermal growth factor receptor 2.

### CCNB1 upstream miRNA prediction and analysis

**Figure 4:**
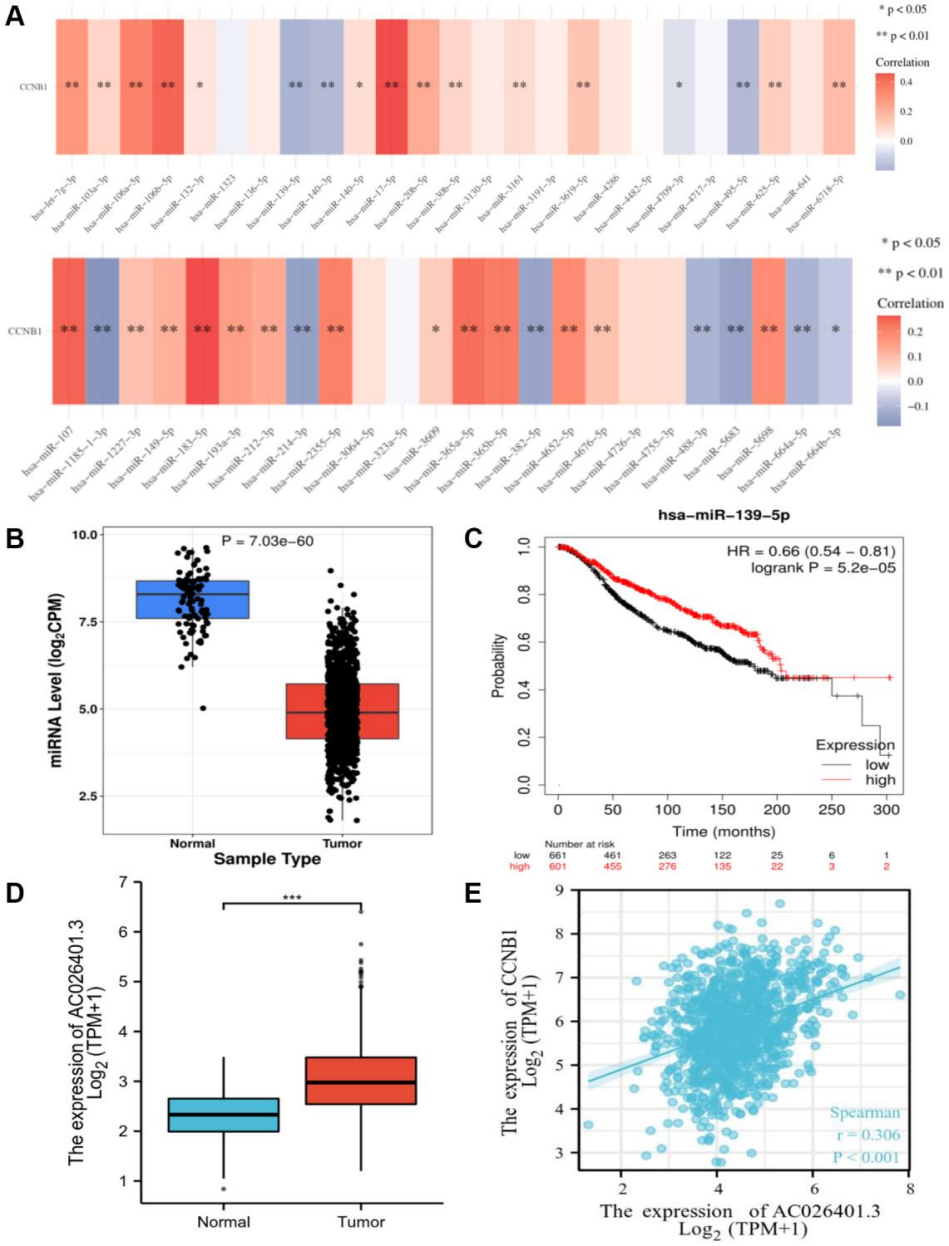
**Identification of miR-139-5p as a potential upstream miRNA of CCNB1, and AC026401.3 as a potential upstream lncRNA of miR-139-5p.** (**A**) The expression correlation between predicted miRNAs and CCNB1 in BC. (**B**) The expression of miR-139-5p in BC and control normal samples was determined. (**C**) The prognostic value of miR-139-5p in BC assessed plotter. (**D**) The expression of AC026401.3 in BC and control normal samples was determined. (**E**) Between CCNB1 and AC026401.3, there was a substantial positive association.

The role of ncRNAs in gene regulation has increasingly been known. To determine whether CCNB1 was influenced by certain ncRNAs, we first anticipated upstream miRNAs that may potentially bind to CCNB1 and eventually discovered 24 miRNAs. There should be a negative association between miRNA and CCNB1 based on miRNA’s action mechanism in the control of target gene expression. In BC, CCNB1 had the strongest negative correlation with miR-139-5p. The expression of miR-139-5p in BC was assessed, as well as its prognostic value. MiR-139-5p was significantly downregulated in BC, as shown in Figure 4B, 4C, and its overexpression was associated to patients’ prognosis. All of these data point to miR-139-5p as the most effective CCNB1 regulating miRNA in BC.

### Upstream lncRNAs of AC026401.3 prediction and analysis

By using starBase database, the upstream lncRNAs of miR-139-5p were projected. A total of 75 lncRNAs have been predicted. GEPIA was used to assess the expression levels of these lncRNAs in BC. Only RP11-553L6.5 was highly elevated and showed prognostic significance in BC when compared to normal controls, as well as being strongly related with CCNB1. According to the competing endogenous RNA (ceRNA) theory, lncRNA can improve mRNA expression by binding to shared miRNAs in a competitive manner. As a result, lncRNA and miRNA should have a negative connection, while lncRNA and mRNA should have a positive association. As suggested in Figure 4, compared with normal breast tissue, RP11-553L6.5 was highly expressed in BC (Figure 4D) and had a significant positive correlation with CCNB1 (Figure 4E).

RP11-553L6.5 may be the most potential upstream lncRNAs of the miR-139-5p/CCNB1 axis in BC, based on expression and correlation analyses.

### TMB and DNA methylation of CCNB1

We then calculated the BC TMB associated with CCNB1 for each patient in the TCGA and GTEx databases, and we found that the expression of CCNB1 in luminal A BC cell lines was positively correlated with TMB, *P* < 0.05; while in BC cell lines the expression in luminal B, Her2+, and Basal like BC cell line was not statistically significant (Supplementary Figure 1A). Correlation analysis results of DNA methylation indicating that CCNB1 and DNA methylation have a negative correlation (Supplementary Figure 1B).

### Expression of CCNB1 in different molecular subtyping of BC

**Figure 5:**
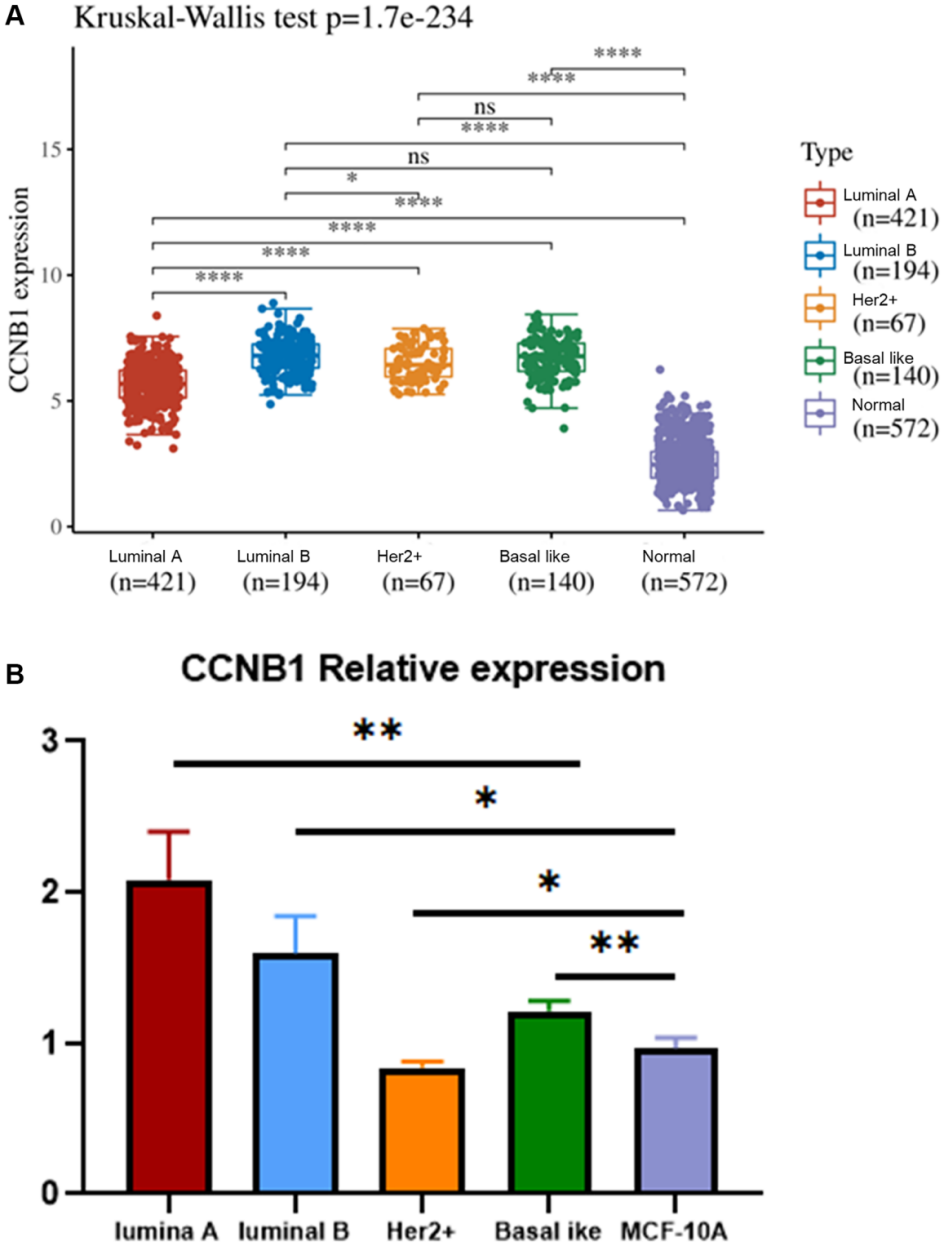
**Expression of CCNB1 in different molecular subtyping of BC.** (**A**) The relative expression of CCNB1 in luminal A, luminal B, Her2+, basal like and MCF 10A cell lines. CCNB1 expression was higher in the four molecular subtyping of BC (luminal A, luminal B, Her2+, basal like) than in the normal group. (**B**) Compared with the MCF 10A cell line, CCNB1 was up-regulated in luminal A, luminal B and basal-like cell lines, but down-regulated in Her2+ cell line, as verified by qRT-PCR. ^*^p < 0.05, ^**^p < 0.01, ^***^p < 0.001. Abbreviation: BC: breast cancer.

We use TCGA and GTEx database to classify BC and normal cases into luminal A, luminal B, Her2+, basal like and normal groups. We found that the expression of CCNB1 in the four molecular subtyping (luminal A, luminal B, Her2+, basal like) of BC was higher than that in the normal group (Figure 5A).

### qRT-PCR

The expression of CCNB1 in luminal A (MCF-7), Luminal B (BT474), Her2+ (SKBR3), Basal like (MDA-MB-231) BC cell lines and human normal breast epithelial cell lines (MCF-10A) was verified by qRT-PCR.

The results showed that the expression of CCNB1 in the luminal A BC cell line was higher than that in the human normal breast epithelial cell line (Figure 5B).

### Identification of DEGs, function and pathway enrichment analysis

**Figure 6:**
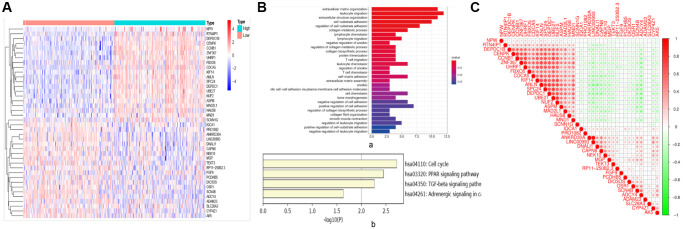
**Related genes and signaling pathways affected by CCNB1.** (**A**) DEGS: heat map. According to the different expression levels of CCNB1, they are divided into two groups: high expression (Green in the right half of the image) and low (Pink in the left half of the image) expression. The abscissa is the sample, and the ordinate is the gene name. (**B**) Go and KEGG analysis. (**a**) Go analysis demonstrated that CCNB1 and genes significantly associated with CCNB1 alterations mainly enriched extracellular matrix organization, leukocyte migration and extracellular structure organization. (**b**) The major enriched pathways were those connected with cell cycle, AMPK, PPAR signaling pathway and TGF signaling beta pathway according to the KEGG analysis. (**C**) The genes that are strongly linked to CCNB1.

**Figure 7:**
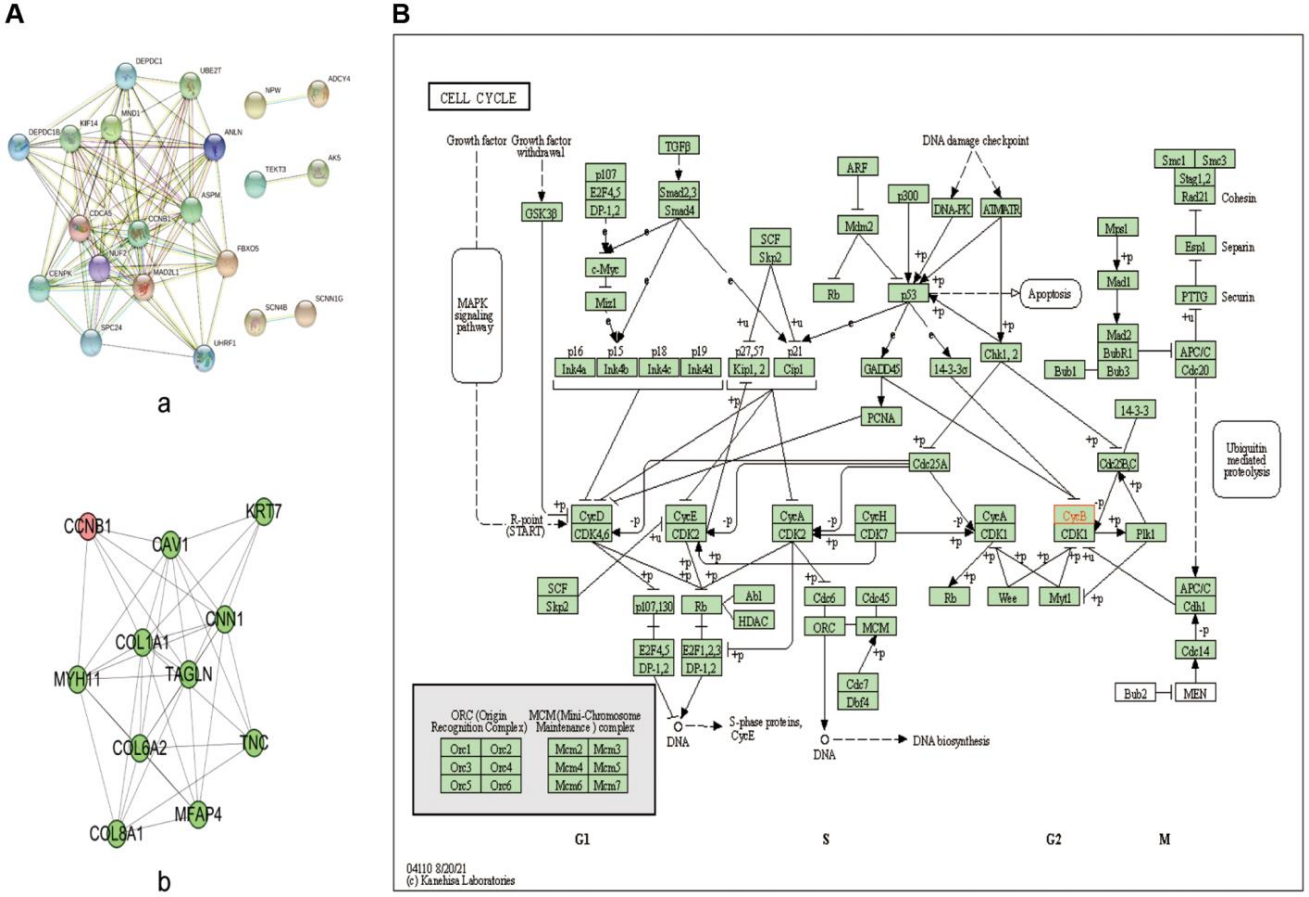
**PPI network and KEGG pathway analysis.** (**A**) PPI network of the significant genes in BC. (**a**) The PPI network was constructed using 40 genes selected from the string web database. (**b**) Construction of CCNB1 subnetwork of PPI. (**B**) KEGG pathway analysis; CCNB1 are involved in the cell cycle. Abbreviations: DEGs: differentially expressed genes; GO: Gene Ontology; KEGG: Kyoto Encyclopedia of Genes and Genomes; PPI: protein-protein interaction.

In this study, 347 BC samples were collected from GSE4922 of GEO database. There were 109 DEGs identified, with 58 up-regulated genes and 51 down-regulated genes. DEGs are visualized on the heat map (Figure 6A). DEGs impacted extracellular matrix organization, leukocyte migration, and extracellular structure organization, according to Go analyses (Figure 6B-a). The cell cycle, PPAR signaling route, and TCF signaling beta pathway were the most enriched in KEGG pathways (Figure 6B-b). Among them, CCNB1 was most enriched in the cell cycle, which was involved in the most significantly enriched signaling pathway in BC tumorigenesis and pathogenesis (Figure 7B).

### Gene mapping and PPI network construction, module analysis of target gene

The interaction between the top 20 genes and the bottom 20 genes was used to perform correlation analysis (Figure 6C). Positive correlation genes with CCNB1: ZNF367, UHRF1, FBXO5, CDCA5, KIF14, ANLN, SPC24, DEPDC1, UBE2T, NUF2, ASPM, MAD2L1, HAUS8, MND1. Negative correlation genes with CCNB1: SCNN1G, IQCA1, PRO1082, ANKRD30A, LINC00993, DNALI1, CAPN8, NEK10, MGP, TEKT3, RP11−250B2.3, FGF9, PCDHB5, DIO3OS, OSR1, SCN4B, ADCY4, ADAM23, SLC28A3, CYP4Z1, AK5. To explore the highly likely correlation between these DEGs, we built a PPI network of DEGs via string and set the confidence score ≥ 0.4 (Figure 7A-a). Finally, we depict the PPI sub network centered on CCNB1 by using Cytoscape (Figure 7A-b).

## DISCUSSION

BC is the second greatest cause of cancer-related mortality in women [13]. Despite progress in screening and testing, it is still urgent to detect the specific and sensitive biomarkers of BC. CCNB1, as a core part of the cyclin family, was found in many cancers [14, 15], especially in BC [16].

In this report, we obtained 1099 BC samples and 292 normal breast samples from TCGA and GTEx for the following study.

We evaluate every gene in Tables 2, 3. We spotted that only CCNB1 satisfies the following conditions: The expression of CCNB1 not only has the most abundant clinical relevance, it is also differentially expressed in BC and normal tissues (also confirmed by our review of the relevant literature [17]) and significant differences in BC patients’ survival rate (Figure 3A, including: OS, overall survival; RFS, recurrence-free survival; DMFS, distant metastasis-free survival). Moreover, CCNB1 can be used as an independent prognostic factor for BC by univariate and multivariate analysis. Because other genes except CCNB1 in Tables 1, 2 do not match the above conditions, we do not show the negative results in the manuscript. After these analyses, we believe that CCNB1 is superior as a breast cancer prognostic biomarker over other genes.

We found widespread, high-level expression of CCNB1 in tumors, and associated with TMB (Figure 1A, 1B). We report that the expression of the CCNB1 was higher in BC than in normal tissues (Figure 1C).

Our study found a positive association between TMB and CCNB1 expression in breast cancer, specifically in luminal A BC. According to research, elevated TMB in cancer cells causes the release of new antigens, which activates a signal cascade that recruits TIL and causes the expression of PD-1/PD-L1 in immune cells and cancer cells [18–21]. The presence of a high TMB indicates the production of a large number of new antigens [22]. Immunosuppressive agents cause T cells to detect novel antigens, making them more likely to attack and kill tumors. Therefore, detection of TMB in BC tissues by CCNB1 can help predict the efficacy of immunosuppressants.

The microenvironment of various cell clusters involved in cancer has become a hot topic in recent years [23]. We found 9 types of tumor invasive immune cells from the expression level of CCNB1 in BC. CCNB1 expression was positively association with the expression of Macrophages M0, Macrophages M1, Dendritic cells activated, T cells follicular helper, T cells CD4 memory activated, while it has a significant negatively correlated with the expression of B cells naive, Mast cells resting, T cells CD4 memory resting and Monocytes. The crosstalk between CCNB1 and tumor infiltrating immune cells in BC indicates that CCNB1 is a potential target for future treatment of BC, although no further relationship can be established in the current study.

The GEO database of GSE4922 was used to acquire 347 samples of BC in this study. It was identified that 10 differentially expressed and survival related genes: BRIP1, CCNB1, CDT1, CENPW, CSRNP3, DIAPH3, ENKD1, LINC00472 (Table 2). The results suggest that high expression of the CCNB1 may adversely affect the survival of BC, which can be used as an independent factor for predicting prognosis through survival and independent prognostic analysis (Figure 3A, 3B). Further analysis of the clinical characteristics related to CCNB1 showed that it is highly expressed in ER (−), lymphnode (+), and p53 (+) groups, respectively (Figure 3C). ER (−), lymphnode (+), and p53 (+) were all adverse prognostic factors for BC patients, indicating that CCNB1 expression was a very unfavorable factor for BC patients. There may be crosstalk between ER and p53 mediated pathways. ERα and p53 play a key roles in BC tumorigenesis [24]. ERα hyperplasia increases the initiation and progression of BC [25], and tumor suppressor p53 is involved in several cell processes, including cell cycle regulation, apoptosis, aging and differentiation [26]. The presence of wild-type p53 in ER (+) BC is a major factor of both positive and favorable treatment response [27]. Patients with overexpression of p53 had shorter overall survival than those without accumulation of p53, regardless of tumor size, lymph node metastases, or diagnosis age [28].

It has previously been reported that ncRNAs, including as miRNAs and lncRNAs, play a role in gene regulation by communicating with one another via the ceRNA mechanism [29, 30]. CCNB1 was the most significantly negatively correlated with miR-139-5p in BC (Figure 4A). After correlation analysis, expression analysis, and survival analysis of miR-139-5p, miR-139-5p was confirmed to be the most plausible upstream tumor suppressive miRNA of CCNB1 (Figure 4A–4C). It has been reported in the literature that decreased miR-139-5p can enhance the metastasis and tumor invasiveness of hepatocellular carcinoma cells and breast cancer [31, 32].

The putative lncRNAs of the miR-139-5p/CCNB1 axis should be carcinogenic lncRNAs in BC, according to the ceRNA hypothesis [22]. Following that, upstream lncRNAs of the miR-139-5p/CCNB1 axis were projected. By using expression analysis and correlation analysis, the most potential upregulated lncRNAs AC026401.3 was identified (Figure 4D, 4E). Glycolysis based AC026401.3 have important value in survival prediction of renal cell carcinoma patients [23], but their specific roles in tumors are unclear and deserve further exploration [33].

AC026401.3/miR-139-5p/CCNB1 axis was identified as potential regulatory pathways in BC (Figure 4).

In BC, we calculated the TMB associated with CCNB1, and we found that the expression of CCNB1 in luminal A BC cell lines was positively correlated with TMB (Supplementary Figure 1A), while negative correlated with DNA methylation (Supplementary Figure 1B). DNA methylation plays a critical role in cancer formation. As an epigenetic change, DNA methylation shows that CCNB1 is a more suitable cancer risk biomarker [34, 35].

We found that the expression of CCNB1 in the four molecular subtyping (luminal A, luminal B, Her2+, Basal like) of BC was higher than that in the normal group (Figure 5A). We then verified this finding by qRT-PCR. Except for Her2+ cell line, the expression of CCNB1 in the other three cell lines (luminal A, luminal B and basal like cell lines) was higher than in human normal breast epithelial cell line, with CCNB1 expression in luminal A cell line being the most significant. (Figure 5B). Considering the high TMB of CCNB1 in luminal A BC, the possibility of CCNB1 as a specific target in luminal A BC increases. But even so, this requires our follow-up experimental verification.

We carried out the related gene analysis of CCNB1 and established a PPI network, and also carried out GO and KEGG analysis (Figures 6, 7). Go analysis showed that related gene analysis of CCNB1 mainly enriched extracellular matrix organization, leukocyte migration and extracellular structure organization. KEGG signaling pathway is abundantly expressed in cell cycle, PPAR signaling pathway, and TCF signaling pathway. The above results suggest that CCNB1 is involved in the pathogenesis and development of tumors [36].

However, there are some limitations in this study. On the one hand, our results need further verification in BC tissues through experimental and clinical studies and further functional analysis and mechanistic studies are significant. On the other hand, we cannot verify its specific biological function and find out the exact signaling pathway.

## CONCLUSION

Our results provide a systematic bioinformatics study of CCNB1, which might play a key role in the initiation, progression, and prognosis of BC. In BC, CCNB1 may serve as a poor prognostic marker and therapeutic target in BC patients. CCNB1 regulates immune cell invasion in the BC microenvironment, that it could be a therapeutic target for controlling the anti-tumor immune response. We discovered an upstream regulatory mechanism of CCNB1 in BC, namely AC026401.3/miR-139-5p/CCNB1 axis. We found that CCNB1 may be the core gene related to BC, it shows that this study can predict the development of BC, understand the mechanism of its development, and even provide useful evidence for genome individualized treatment.

## Supplementary Materials

Supplementary Figure 1
