# Correction for: High expression of CCNB1 driven by ncRNAs is associated with a poor prognosis and tumor immune infiltration in breast cancer

**DOI:** 10.18632/aging.206137

**Published:** 2024-10-15

**Authors:** Hongtao Fu, Kun Li, Shui Wang, Yuming Li

**Affiliations:** 1 Department of Breast Surgery, Jiangsu Province Hospital, The First Hospital Affiliated Hospital with Nanjing Medical University, Nanjing 210029, China; 2 Department of Emergency, Changsha Central Hospital, The Affiliated Changsha Central Hospital of Hengyang Medical School, University of South China, Changsha 410004, China; 3 Department of Traditional Chinese Medicine and Western Medicine, Hunan Cancer Hospital, The Affiliated Cancer Hospital of Xiangya School of Medicine, Central South University, Changsha 410006, China

**Keywords:** biomarker, bioinformatics analysis

**This article has been corrected:** The authors corrected the order of the affiliations and correct order is listed below:

**Hongtao Fu^1,2^, Kun Li^3^, Shui Wang^2^, Yuming Li^1^**

^1^Department of Traditional Chinese Medicine and Western Medicine, Hunan Cancer Hospital, The Affiliated Cancer Hospital of Xiangya School of Medicine, Central South University, Changsha 410006, China

^2^Department of Breast Surgery, Jiangsu Province Hospital, The First Hospital Affiliated Hospital with Nanjing Medical University, Nanjing 210029, China

^3^Department of Emergency, Changsha Central Hospital, The Affiliated Changsha Central Hospital of Hengyang Medical School, University of South China, Changsha 410004, China

