# Supplementary material for: High expression of CCNB1 driven by ncRNAs is associated with a poor prognosis and tumor immune infiltration in breast cancer
Source: Aging (Albany NY). 2022 Aug 29;14(16):6780–95. doi: 10.18632/aging.204253 (PMC9467392; doi:10.18632/aging.204253)
Supplement: Supplementary Figure 1 [file aging-14-204253-s001.pdf]

SUPPLEMENTARY FIGURE

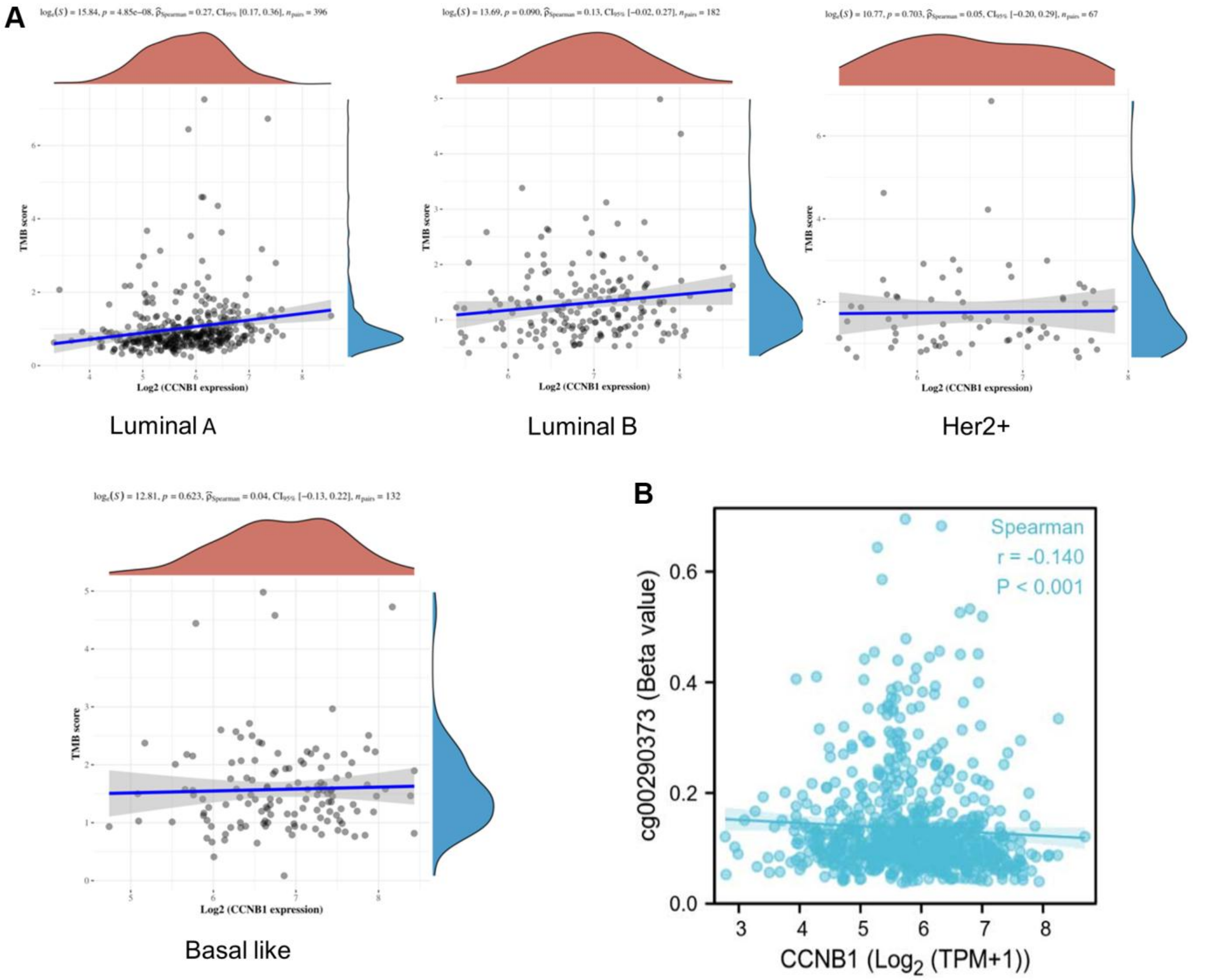

Supplementary Figure 1. Relative expression of CCNB1 in TMB, DNA methylation and luminal A BC cell lines. (**A**) The expression of CCNB1 was positively correlated with TMB in luminal A BC cell line, $P < 0.05$, but not in luminal B BC cell line, Her2+ (i.e., Her2 enrich), Basal like BC cell line, $P > 0.05$. (**B**) Correlation analysis results of DNA methylation indicating that CCNB1 with DNA methylation have a negative correlation. Abbreviations: TMB: tumor mutation burden; BC: breast cancer.
